# Spatial mRNA Expression and Response to Fasting and Refeeding of Neutral Amino Acid Transporters *slc6a18* and *slc6a19a* in the Intestinal Epithelium of *Mozambique tilapia*

**DOI:** 10.3389/fphys.2018.00212

**Published:** 2018-03-13

**Authors:** Zenith Gaye A. Orozco, Satoshi Soma, Toyoji Kaneko, Soichi Watanabe

**Affiliations:** Department of Aquatic Bioscience, Graduate School of Agricultural and Life Sciences, The University of Tokyo, Tokyo, Japan

**Keywords:** *slc6a18*, *slc6a19a*, neutral amino acid transporter, fasting, refeeding, intestinal amino acid transporter, *Mozambique tilapia*, nutrient absorption

## Abstract

The mRNA expressions of the epithelial neutral amino acid transporters *slc6a18* and *slc6a19a* in the five segments (HL, PMC, GL, DMC, and TS) of the intestine of *Mozambique tilapia*, and their responses to fasting and refeeding were investigated for a better understanding of the functional and nutritional characteristics of *slc6a18* and *slc6a19a*. Although both *slc6a18* and *slc6a19a* were expressed mainly in the intestine, these genes showed opposing spatial distributions along the intestine. The *slc6a18* was mainly expressed in the middle (GL) and posterior (DMC and TS) intestines, while *slc6a19a* was specifically expressed in the anterior intestine (HL and PMC). Large decreases of amino acid concentrations from the HL to GL imply that amino acids are mainly absorbed before reaching the GL, suggesting an important role of *slc6a19a* in the absorption. Moreover, substantial amounts of some neutral amino acids with the isoelectric point close to 6 remain in the GL. These are most likely the remaining unabsorbed amino acids or those from of amino acid antiporters which release neutral amino acids in exchange for uptake of its substrates. These amino acids were diminished in the TS, suggesting active absorption in the posterior intestine. This suggests that *slc6a18* is essential to complete the absorption of neutral amino acids. At fasting, significant downregulation of *slc6a19a* expression was observed from the initial up to day 2 and became stable from day 4 to day 14 in the HL and PMC suggesting that *slc6a19a* expression reflects nutritional condition in the intestinal lumen. Refeeding stimulates *slc6a19a* expression, although expressions did not exceed the initial level within 3 days after refeeding. The *slc6a18* expression was decreased during fasting in the GL but no significant change was observed in the DMC. Only a transient decrease was observed at day 2 in the TS. Refeeding did not stimulate *slc6a18* expression. Results in this study suggest that Slc6a18 and Slc6a19 have different roles in the intestine, and that both of these contribute to establish the efficient neutral amino acid absorption system in the tilapia.

## Introduction

Amino acids are vital nutrients and are important regulators of key metabolic pathways and physiological processes in organisms. Amino acids are acquired mainly from dietary proteins. Ingested proteins are digested into amino acids in the gastrointestinal tract by various digestive enzymes. Amino acids, in the form of free amino acids and oligopeptides, are transported from the lumen across the intestinal epithelial cells into the blood stream by the amino acid transporters present in the apical and basolateral membranes of the intestinal epithelial cells (Mailliard et al., 1995; Nordrum et al., 2000). Therefore, amino acid transporters are responsible for the continuous supply of amino acids to all tissues and maintaining amino acid homeostasis in the body (Bröer, 2008b). Despite many studies on amino acid absorption, the functions and characteristics of amino acid transporters are not fully understood.

In fish, while many nutritional studies are focused on the factors affecting nutrient utilization, growth rates, disruption of other physiological processes, lesser studies are carried out on the molecular level including the role of amino acid transporters in the absorption and amino acid homeostasis in the body. So far, most of the studies reported on amino acid transporters in fish have been focused on peptide transporter Slc15a1. Cloning and functional characterization of Slc15a1 has been carried out in species such as zebrafish *Danio rerio* (Verri et al. 2003), Atlantic cod *Gadus morhua* (Rønnestad et al., 2007), European sea bass *Dicentrarchus labrax* (Terova et al., 2009), Atlantic salmon *Salmo salar* (Rønnestad et al., 2010), killifish *Fundulus heteroclitus macrolepidotus* (Bucking and Schulte, 2012), grass carp *Ctenopharyngodon idella* (Liu et al., 2013), and yellow perch *Perca flavescens* (Kwasek et al., 2012). Previous studies showed variations in the tissue distribution of Slc15a1 in different fish species (Verri et al., 2003; Terova et al., 2009; Rønnestad et al., 2010; Ahn et al., 2013; Orozco et al., 2017). It has also been shown that Slc15a1 is affected by changes in environmental (Bucking and Schulte, 2012; Rimoldi et al., 2015) and nutritional (Hakim et al., 2009; Terova et al., 2009; Bakke et al., 2010; Ostaszewska et al., 2010; Bucking and Schulte, 2012; Koven and Schulte, 2012; Orozco et al., 2017) conditions. Moreover, the response of Slc15a1 to the change in nutritional condition (e.g., availability of food, dietary treatments) varies among species. For example, the Slc15a1 expression patterns and response time to fasting differ among killifish, sea bass and *Mozambique tilapia* (Hakim et al., 2009; Bucking and Schulte, 2012; Orozco et al., 2017). These variations were suspected to be due to distinct morphological and phenotypic characteristics of each species. In fact, the gastrointestinal characteristic of fish is greatly influenced by their dietary preference; that is, herbivore, omnivore, and carnivore (Lassiter and Edwards, 1982).

Several studies were conducted on free amino acid transporters in fish. Complete sequences of neutral amino acid transporter *slc6a19* has been reported in sea bass *D. labrax* (Margheritis et al., 2013, 2016; Rimoldi et al., 2015), and zebrafish (*D. rerio*) (Tian et al., 2015). Rimoldi et al. (2015) reported that *slc6a19* was expressed in the intestine of sea bass with the highest expressions in segments 8 and 9, followed by segments 1–7 and 10. Rimoldi et al. (2015) also examined the effect of salt-enriched diet on *slc6a19* in the intestine of sea bass by adding 3% NaCl to 10% fishmeal diet. Their results showed that the expressions of *slc6a19* in the anterior and posterior intestine were not affected by the treatment. Nitzan et al. (2017) reported that slc6a19 was influenced by time after feeding. Their results showed that higher expression of *slc6a19* was observed in the middle as compared to the anterior and posterior intestines at 6 h after feeding of freshwater-reared *M. tilapia*. They also studied the effect of salinity on *slc6a19, slc3a1*, and *slc7a9*. Their results showed that only *slc3a1* and *slc7a9* were influenced by salinity (Nitzan et al., 2017). In zebrafish, expressions of amino acid transporters *slc6a14* (ATB^0, +^) and *slc6a19* (B^0^AT1) were decreased at first, then increased, and finally decreased again during fasting. Their expressions were high at 24–96 h of fasting suggesting that protein utilization may occur during these period (Tian et al., 2015). On the other hand, *slc1a5* (ASCT2) expression increased after fasting for 3–6 h and began to decrease after 12 h of fasting (Tian et al., 2015).

In fish, deficiency in neutral amino acids leads to the development of diseases, reduced protein utilization and growth rate, and disruption of other physiological activities (Sveier et al., 2001; Craig and Moon, 2013; Belghit et al., 2014). These reports suggested that neutral amino acid is of key importance to maintain adequate living conditions in organisms. Therefore, clarification of neutral amino acid absorption is essential to understand amino acid absorption mechanisms in the body. Since the varied distribution of neutral amino acid transporter in the intestine has been reported in other species (i.e., *slc6a19*), precise investigation of spatial distribution of neutral amino acid transporters may provide important information to understand the dynamics of neutral amino acid absorption. We then conducted preliminary studies on solute carrier 6 (Slc6) transport family containing neutral amino acid transporters, and found highly-expressed *slc6a18* and *slc6a19a* in the intestine. We hypothesize that these two transporters are the major players in the absorption of neutral amino acid in the tilapia intestine.

In the present study, the expressions of *slc6a18* and *slc6a19a* genes that encode the neutral amino acid transporters Slc6a18 and Slc6a19, were investigated in the intestine of *M. tilapia*. Only one study has been reported on *slc6a19* in this species (Nitzan et al., 2017), but the complete code for *slc6a19* in tilapia has not been reported so far. Also, unlike in seabass where a detailed examination of *slc6a19* expressions in the intestinal sections was conducted (Rimoldi et al., 2015), a more thorough distribution studies are still needed in tilapia. Tilapia has a long intestine arranged in a complex manner involving multiple loops and coils, a characteristic common in herbivorous and omnivorous fish which may reflect their adaptability in diet (Smith et al., 2000). Therefore, a more detailed examination of spatial distribution is important for a better understanding of the functions and characteristics of these transporters. Meanwhile, there is still no study on *slc6a18* in fish. Here, the distribution of *slc6a18* and *slc6a19a* in the tissues of *M. tilapia* was examined. Zonation in the five segments of the intestine was then investigated. The responses of intestinal *slc6a18* and *slc6a19* to the changes in nutrient condition induced by fasting and refeeding were examined. Results of the present study may contribute to a better understanding of the functional and nutritional characteristics of neutral amino acid transporters Slc6a18 and Slc6a19 in fish.

## Materials and methods

### Fish and experimental design

*M. tilapia Oreochromis mossambicus* (100–250 g) were maintained in a 3,000-l tank with recirculating freshwater at 25°C. Tilapia were fed to satiation once a day on commercial carp chow (Nihon Haigo Shiryo, Kanagawa, Japan). Prior to experiments, fish were transferred and acclimatized for 1 week in experimental tanks with recirculating freshwater maintained at 25°C.

The effect of fasting and refeeding on *slc6a18* and *slc6a19a* expressions were investigated in this study. In the fasting experiment, fish were reared without feeding for 14 days. Fish (*n* = 6 each) were sampled before the start of fasting as controls (day 0, 30 min after feeding), and at 1, 2, 4, 7, and 14 days after the last feeding (days 1, 2, 4, 7, and 14). A separate experiment was conducted to investigate the effect of refeeding on expressions of these genes. In this experiment, fish were first starved for 14 days and fed again on a daily basis (refeeding experiment). Fish were fed to satiation once a day at 10:00 in the morning upon resumption of feeding. Fish (*n* = 6 each) were sampled before fasting (controls), at 14 days after the last feeding, and at 1 day and 3 days after refeeding. Sampling time (13:00 to 15:00) was the same for every collection of samples for fasting and refeeding experiments. For the fasting and refeeding experiments, fish were reared individually in the same water recirculating system to minimize the effect of aggressive behavior and social hierarchies. For all samplings, the weight and the standard length of fish were measured. Stomach content at initial time point (30 min after feeding) was weighed and standardized with body weight to confirm the amount of feed intake under experimental condition (44.78 ± 4.26 g stomach content/kg body weight). For the other time points in the fasting experiment, the availability of ingested feed in the stomach and intestine was checked by visual observation. Fish were anesthetized with 2-phenoxyethanol (0.1%) prior to dissection and tissue collection. These procedures were approved by the Committee for the Care of Laboratory Animals in the Graduate School of Agricultural and Life Sciences at the University of Tokyo. Processing of tissue samples and analyzes of gene expressions of *slc6a18* and *slc6a19a* were carried out as described below.

### Tissue sampling and cDNA synthesis

Tissue distribution analysis for *slc6a18* and *slc6a19a* were carried out in the brain, gill, heart, liver, kidney, esophagus, stomach, intestine, rectum, muscle, and skin of tilapia. The intestine was divided into five segments as described in Smith et al. (2000): the hepatic loop (HL), proximal major coil (PMC), gastric loop (GL), distal major coil (DMC), and terminal segment (TS). The collected tissues were washed with 0.01 M phosphate-buffered saline (PBS). Tissues were stored in RNA extraction reagent (ISOGEN, Nippon Gene, Tokyo, Japan) at −80°C until analysis. The total RNA extraction was carried out according to the manufacturer's instruction. Total RNA (2 μg for each sample) was treated with DNase I (Roche Diagnostics, Basel, Switzerland) and reversed-transcribed to single stranded cDNA using the high capacity RNA-to-cDNA Kit (Thermo Fisher Scientific, Waltham, MA) or SMARTer RACE 5′/3′ kit (Takara, Otsu, Japan) following the manufacturers' instructions. For the fasting and refeeding experiments, only the five segments of the intestine (HL, PMC, GL, DMC, and TS) were analyzed for gene expressions. For histological analysis, anterior and posterior parts of the intestine were fixed in 4% paraformaldehyde (PFA) in 0.1 M phosphate buffer for 16 h, and stored in absolute methanol at −20°C until use.

### cDNA cloning

Degenerate PCRs for *slc6a18* and *slc6a19a* were carried out using primer pairs listed in the Table 1. The PCR products were ligated into pGEM T-easy (Promega, Madison, WI) and sequenced by a DNA sequencer, ABI PRISM 3130xl (Thermo Fisher Scientific). Sequence data were analyzed with ATGC software (Genetyx, Tokyo, Japan). After determination of the partial cDNA sequences of *slc6a18* and *slc6a19a*, 3′- and 5′-rapid amplification of cDNA ends (RACE) were carried out to extend sequence information at 3′ and 5′ ends with adaptor primers in SMARTer RACE 5′/3′ kit and gene-specific primers for *slc6a18* and *slc6a19a* (Table 1). Topology prediction for the deduced amino acid sequence of tilapia *slc6a18* and *slc6a19a* were performed with the software TMHMM (www.cbs.dtu.dk/services/TMHMM/). Phylogenetic analysis among slc6a15-20 in various vertebrates was done with a ClustalW software (Ver. 1.83, http://clustalw.ddbj.nig.ac.jp/index.php?lang=en).

**Table 1:** Sequence (5′ → 3′) of primers used in the experiments.

	**Name**	**Sequence (5′ → 3′)**
Degenerate PCR	slc6a18-df	GCN TGG RTN ATG TGG TA
	slc6a18-dr	TCI GTR AAN ACD ATR AAN GC
	slc6a19-df	AAY GTI TGG MGN TTY CCN TA
	slc6a19-dr	ATR AAI GCI ARN CCN GTN CC
5′-RACE	Omslc6a18-5′r	CAT TTA GTG GGC AGG TGC TCC AC
	Omslc6a19a-5′r	ATG GCA AAC TCT AAG TGC AGC AGC
3′-RACE	Omslc6a18-3′f	AGA GTC TGT CCC TGA GGG AGT GTG
	Omslc6a19a-3′f	AAC TAC GAC CAG GTC CTG ACC AAC
Quantitative PCR	Omslc6a18-qf	CAT GGG AAA GGC TGT GTA TG
	Omslc6a18-qr	GTA GCT CCA GGC AGA GTG
	Omslc6a19a-qf	ACT GGA GTT GGC ATT GCA TC
	Omslc6a19a-qr	TGT GCT GTT AGC GTT GAG AG
	Omef1a-qf	TGC GGA GGA ATC GAC AAG AG
	Omef1a-qr	AGG AGC CCT TTC CCA TCT CA
*In situ* hybridization	Omslc6a18-pf	AAC ATC CTG GCT CTG ACG AA
	Omslc6a18-pr	ATC TGG ATT TTT GCG GTG TG
	Omslc6a19a-pf	CAC AAG ATT GCG ACT GCT GA
	Omslc6a19a-pr	CCT CAG GTC AGG ATC AGT GC

### Quantitative real-time PCR analysis

The cDNA samples were subjected to quantitative real-time PCR (qPCR) using the LightCycler 480 System II (Roche Diagnostics, Basel, Switzerland) with LightCycler FastStart DNA Master PLUS SYBR Green I (Roche Diagnostics) with 45 cycles of amplification. Analyses were conducted according to the manufacturer's instructions. Gene expressions were quantified using the Second Derivative Maximum method. To confirm the specificity of qPCR, melting curve analyses were performed. The gene expressions of *slc6a18* and *slc6a19a* were analyzed in all tissue samples of *M. tilapia* for tissue distribution analysis (the brain, gill, heart, liver, kidney, esophagus, stomach, intestine (HL, PMC, GL, DMC, TS), rectum, muscle, and skin) with primer sets (Table 1). Forward (*slc6a18 and 6a19a*) or reverse (*ef1a*) primers for qPCR were designed at the exon-intron boundaries of each genes to amplify cDNA derived from mature mRNA specifically. For the fasting and refeeding experiments, *slc6a18* expression was analyzed only in the GL, DMC, and TS based on the results of the tissue distribution analysis. The *slc6a19a* expressions were analyzed in the HL and PMC. Abundance of transcripts encoding elongation factor 1α (*ef1a*) was also measured as an internal standard with a primer set (Table 1). Expression data of internal standard gene in this study are shown in supplementary materials (Figures S1–S3). Standard dilution series with plasmids containing target sequences (1.0 × 10^1^ to 1.0 × 10^6^ copies/μl) was prepared. Amplification of the standard was observed from 10 to 35 cycles. Successful reverse transcription of cDNA templates obtained from tissues was confirmed by constant amplification of *ef1a* among all samples. The efficiency of the amplification reaction of the *ef1a, slc6a18*, and *slc6a19a* were 1.766, 1.923, and 1.885, respectively.

### Total protein and amino acid analysis in the intestinal chyme

The total protein and free amino acid concentrations in the intestinal chyme were examined at 6 h after feeding in the HL (the most proximal part of the intestine), GL (the intermediate part) and TS (the most distal part). Upon dissection of fish (*n* = 5), the most posterior part of the terminal segment was tied with a thread to seal and avoid excretion of the chyme. The HL, GL, and TS were then carefully located in the gastrointestinal tract, and the end of each segment were sealed with a thread before cutting off from other segments. The weight of each intestinal segment was measured before and after collecting the total chyme. The total chyme was then centrifuged at 7,000 rpm at 4°C for 5 min to separate the undissolved matters from the intestinal fluid. Total protein concentration of the intestinal fluid was measured using a bicinchoninic acid (BCA) assay kit following manufacturer's protocol (Thermo Fisher Scientific). The intestinal fluid was diluted 100 times and run in triplicate at 562 nm using a microplate spectrophotometer (Multiskan GO, Thermo Fisher Scientific). The undissolved matter collected from total chyme of each segment was washed seven times (preliminary experiments showed that after seven times of washing, dissolved protein in the diluent becomes negligible after seven times of washing) and stored overnight in −70°C bio-freezer. Those samples were lyophilized and then weighed. The weight of dried undissolved matter was subtracted from the weight of total chyme to obtain the total weight of the intestinal fluid. The dry matter was dissolved in 20 mM sodium hydroxide and subjected to protein analysis using the BCA kit. Total protein concentration was estimated using an 8 point standard curved (μg/ml). The measured protein concentration was normalized to the total volume of intestinal fluid and the fish body weight. To neutralize the body size factor, the values of protein abundance in each segment are presented as milligram protein per gram body weight (mg/g).

For the determination of amino acid concentration profiles, protein were precipitated from the intestinal fluid using 10% trichloroacetic acid (TCA) at a ratio of 1:1. Samples were centrifuged to remove precipitates. Samples were then defatted with hexane and the obtained aqueous phase was filtered (0.2 μm membrane filter) before analysis. Amino acid concentration profiles were analyzed by a Hitachi Elite LaChrom HPLC System (Hitachi High Technologies, Tokyo, Japan).

### *In situ* hybridization

Partial cDNA fragments of tilapia *slc6a18* (1165-2112) and *slc6a19a* (2156-3077) were obtained with primer pairs listed in Table 1, and subcloned into the pGEM-T-Easy vector (Promega). After confirmation of sequences, the resulting constructs were used to generate digoxigenin (DIG)-labeled cRNA probes for *slc6a18* and *slc6a19a* detection using DIG RNA Labeling Mix and T7/SP6 RNA polymerases (Roche Diagnostics). Fixed tissues (the PMC and DMC) were dehydrated in ethanol and embedded in paraffin. The sections (10-μm thickness) were digested with proteinase K (Wako, Osaka, Japan) for 15 min at 37°C, then postfixed with 4% PFA for 10 min, and acetylated with 0.25% acetic anhydride in 0.1 M triethanolamine for 15 min. Hybridization was conducted overnight at 55°C with the DIG-labeled sense and antisense probes in hybridization buffer (50% formamide, 5x saline-sodium citrate (SSC), 5x Denhardt's solution, 2 mg/ml yeast RNA, and 30 μg/ml calf thymus DNA). The sections were washed in 5x SSC with 50% formamide for 20 min at 55°C and twice in 2x SSC for 20 min at 55°C. The hybridized probes were visualized using alkaline phosphatase-conjugated anti-DIG Fab fragment (Roche Diagnostics) diluted 1:2000 and 5-bromo-4-chloro-3-indolyl phosphate/nitro blue tetrazolium substrate (Roche Diagnostics). The specificity of hybridization signals was confirmed by hybridization with the sense probe in place of the antisense probe.

### Statistical methods

Data were expressed as means ± S.E.M. Normality and variance were tested using Shapiro-Wilk and Brown-Forsythe tests, respectively. The significant differences in gene expressions among tissues were analyzed by one-way repeated measures analysis of variance (one-way RM ANOVA) with *post-hoc* Tukey HSD to test comparison. The significant differences in amino acid concentrations and gene expressions among intestinal segments and among groups at different time points were tested using one-way analysis of variance (ANOVA) and the Tukey's HSD test for comparison. *P* < 0.05 was considered statistically significant. All tests were examined using Sigma-Plot 13 (Systat Software, San Jose, CA).

## Results

### cDNA cloning

**Figure 1:**
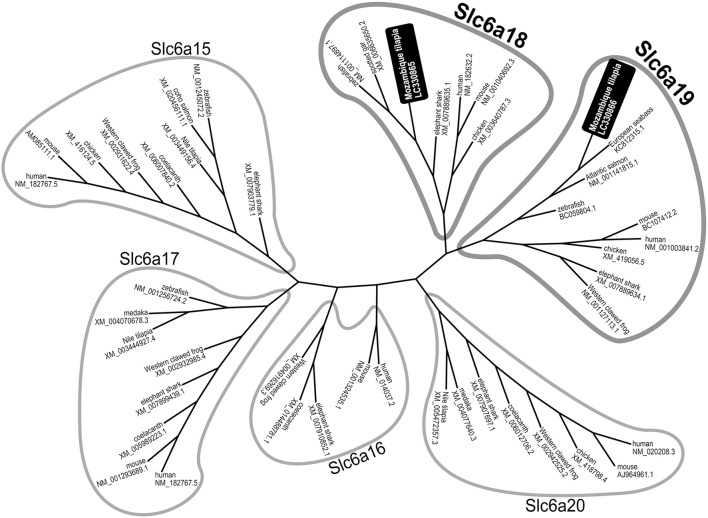
Phylogenetic tree analysis among known Slc6a15-20 family generated by the neighbor-joining method. The deduced amino acid sequences of *slc6a18* and *slc6a19a* identified in this study were distributed to a clade of Slc6a18 and Slc6a19, respectively.

*M. tilapia slc6a18* and *slc6a19a* (GenBank accession nos. LC330865 and LC330866) encode proteins of 617 and 635 amino acids, respectively. According to the topology prediction, tilapia Slc6a18 and Slc6a19a showed conserved structural features among known Slc6a18 and Slc6a19a protein molecules, respectively, in other species (data not shown). According to phylogenetic tree analysis among known Slc6a15–20, the deduced amino acid sequences of *slc6a18* and *slc6a19a* we identified in this study were distributed to a clade of Slc6a18 and Slc6a19, respectively (Figure 1). We also identified another partial sequence similar to *slc6a19* (tentatively referred to as *slc6a19b*) from *M. tilapia*, whose intestinal expression level was more than 10 times lower than *slc6a19a* (data not shown).

### *slc6a18* and *slc6a19a* tissue distribution analysis

**Figure 2:**
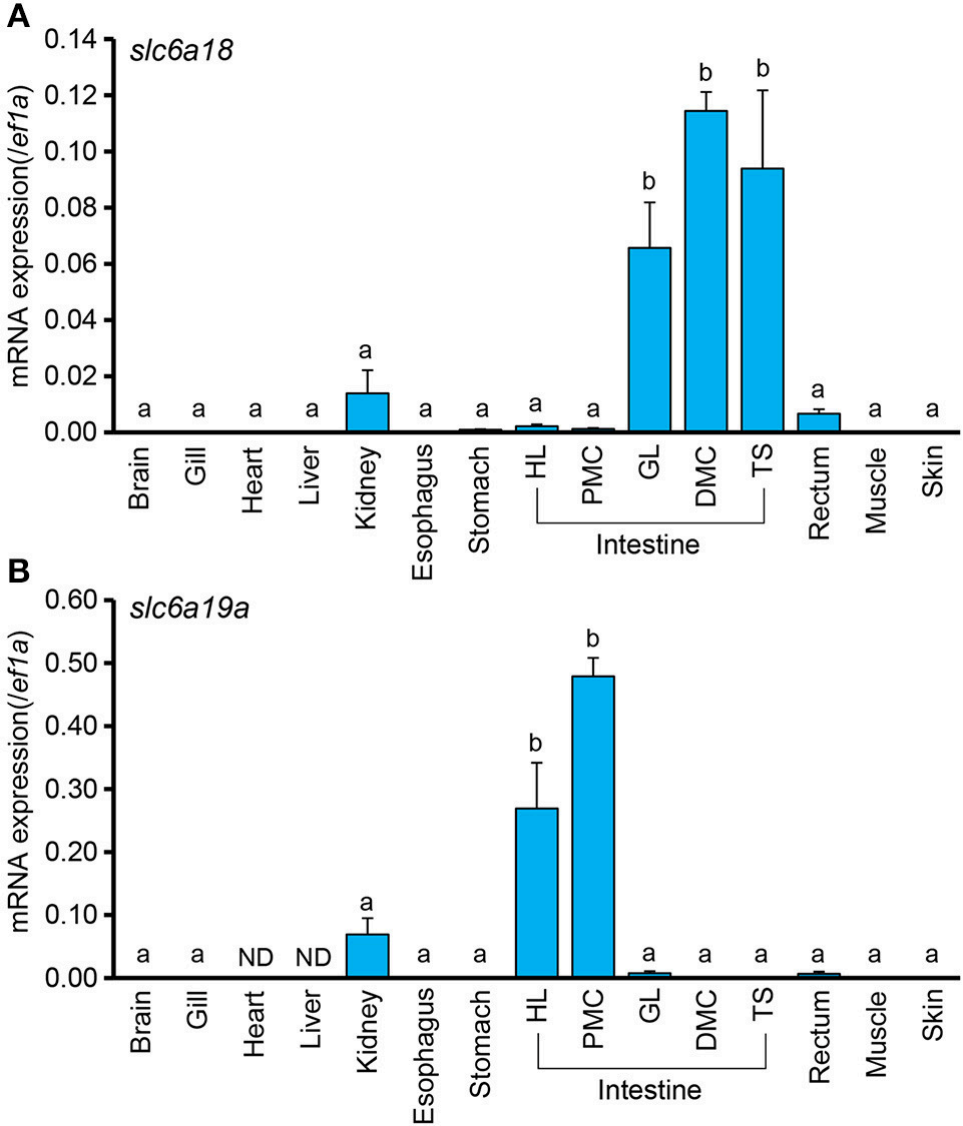
Expressions of *slc6a18* and *slc6a19a* mRNAs in tissues of *Mozambique tilapia* normalized against *ef1a*. Both genes are mainly expressed in the intestine, and poorly or not detected (ND) in other tissues. The *slc6a18* **(A)** is highly expressed in the posterior intestine (GL, PMC, TS) while *slc6a19a* **(B)** is mainly expressed in the anterior intestine (HL and PMC). Values with different letters are significantly different (*P* < 0.05). Error bars indicate the standard error of the mean (SEM).

To examine the tissue distribution of *slc6a18* and *slc6a19a* in *M. tilapia*, mRNA expressions of these genes were analyzed in the brain, gill, heart, liver, kidney, esophagus, stomach, intestine (HL, PMC, GL, DMC, and TS), rectum, muscle and skin. Elongation factor 1 (*ef1a*) was used as internal standard. Both *slc6a18 and slc6a19a* were predominantly expressed in the intestine of *M. tilapia* (Figures 2A,B). Results also showed opposing expression patterns of *slc6a18* and *slc6a19a* in the intestine. The *slc6a18* was mainly expressed in the GL, DMC and TS (middle and posterior parts) while *slc6a19a* was specifically expressed in the HL and PMC (anterior part). These results may indicate spatial functions of these genes in the absorption of amino acids in the intestine. The *slc6a18 and slc6a19a* were also detected in the kidney, but the expression levels were significantly lower than those in the intestine. Both genes were either poorly expressed or not detected in other tissues tested. Based on these results, *slc6a18* expression was analyzed only in GL, DMC and TS while *slc6a19a* expression was analyzed only in the HL and PMC for the fasting and refeeding experiments.

### *In situ* hybridization

**Figure 3:**
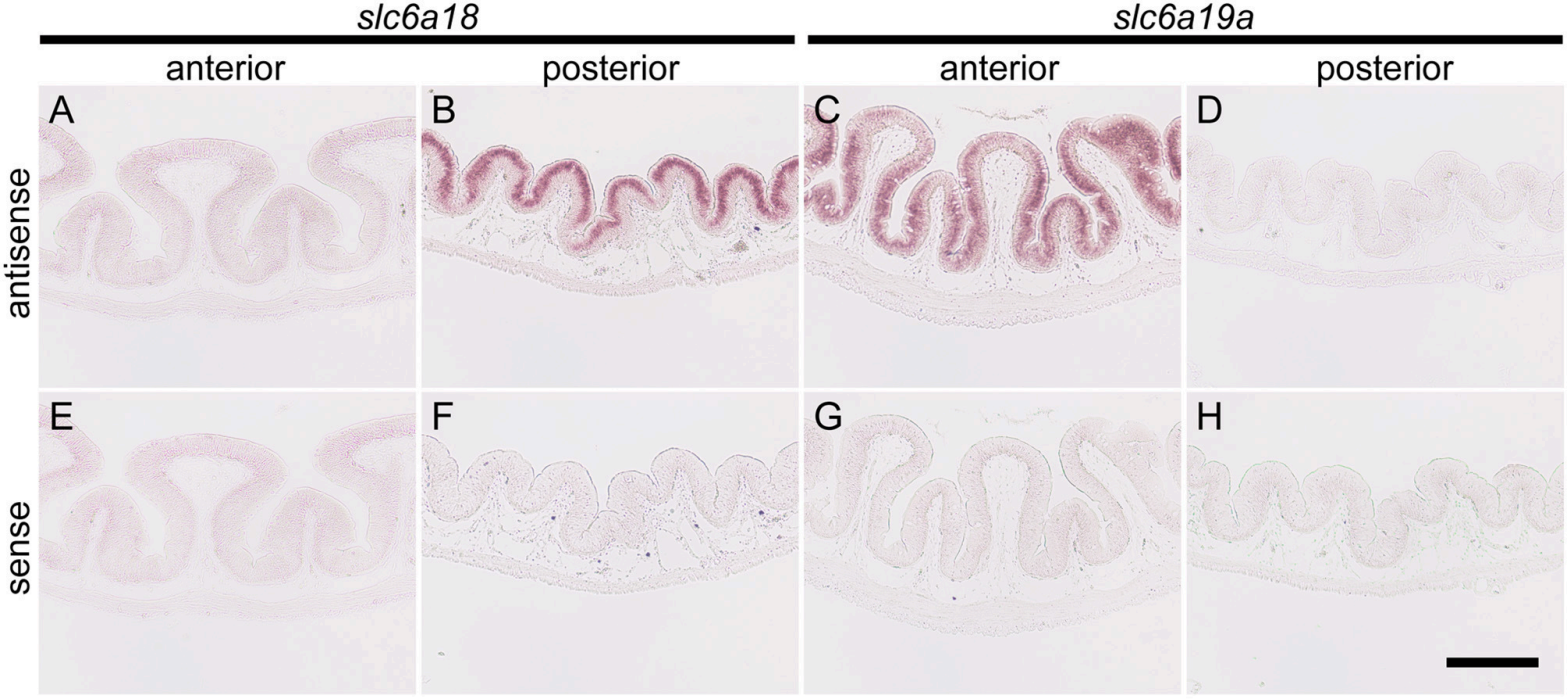
*In situ* hybridization for *slc6a18* and *slc6a19a* in the anterior (PMC) and posterior (DMC) intestine of *Mozambique tilapia*. The *slc6a18* is predominantly expressed in the absorptive epithelial cells of the posterior intestine **(B)** and no signal was observed in anterior intestine **(A)**. The *slc6a19a* was mainly expressed in the anterior intestine **(C)** while no signal was observed in the posterior intestine **(D)**. Specificity of the detection method was confirmed in the anterior and posterior intestines with the sense probes of *slc6a18* **(E,F)** and *slc6a19a* **(G,H)**. Bar: 200 μm.

Intensive hybridization signals for *slc6a18* were specifically detected in the absorptive epithelial cells of the posterior intestine (DMC), whereas those cells of the anterior intestine showed no signals (Figures 3A,B). Conversely, the expression of *slc6a19a* was only observed in the absorptive epithelia of the anterior intestine (PMC), but not in those of the posterior intestine (Figures 3C,D). The specificities of the hybridization signals for *slc6a18* and *slc6a19a* observed in the intestine were confirmed by the control experiments where the antisense probes were replaced with the sense probes (Figures 3E–H).

### Total protein and amino acid concentration profiles

**Figure 4:**
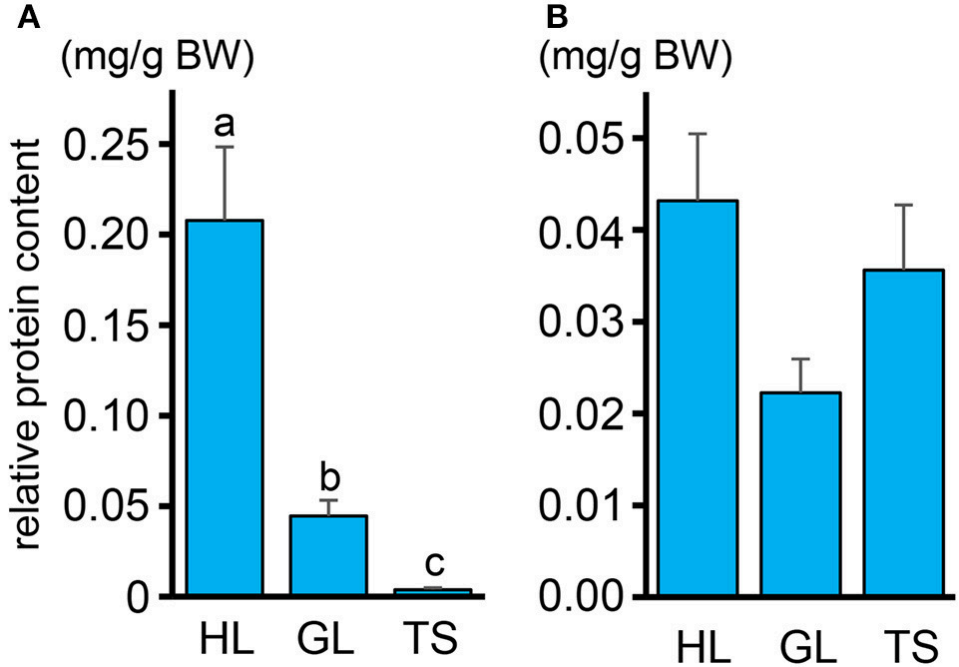
**(A)** Relative protein contents in the intestinal fluid expressed in milligram of protein (normalized against total weight of intestinal fluid in each segment) per gram of fish body weight (mg/g) in the HL, GL, and TS. **(B)** Protein contents in the solid matter from the chyme expressed in weight of protein (mg) relative to the total weight (g) in the HL, GL, and TS. Values with different letters are significantly different (*P* < 0.05). Error bars indicate the standard error of the mean (SEM).

**Figure 5:**
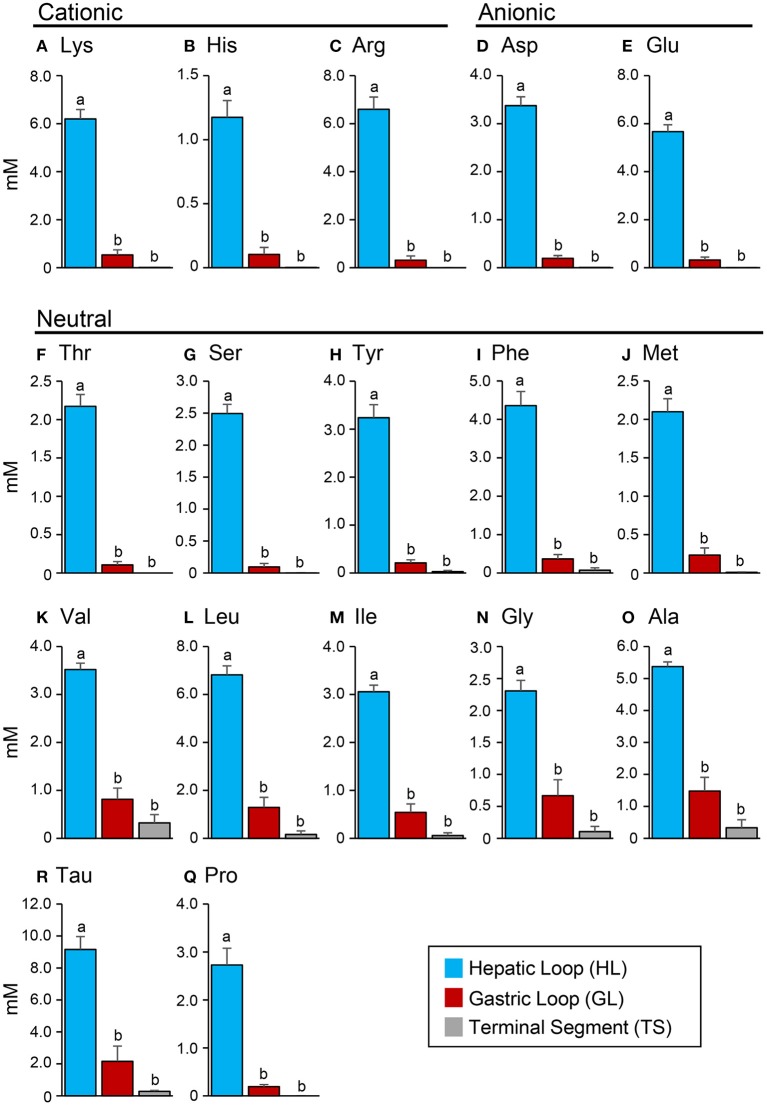
**C**oncentrations (mM) of cationic amino acids **(A–C)**, anionic amino acids **(D,E)** and neutral amino acids **(F–Q)** in the hepatic loop (HL), gastric loop (GL), and terminal segment (TS) of the *Mozambique tilapia* intestine at 6 h after feeding. Values with different letters are significantly different (*P* < 0.05). Error bars indicate the standard error of the mean (SEM).

**Figure 6:**
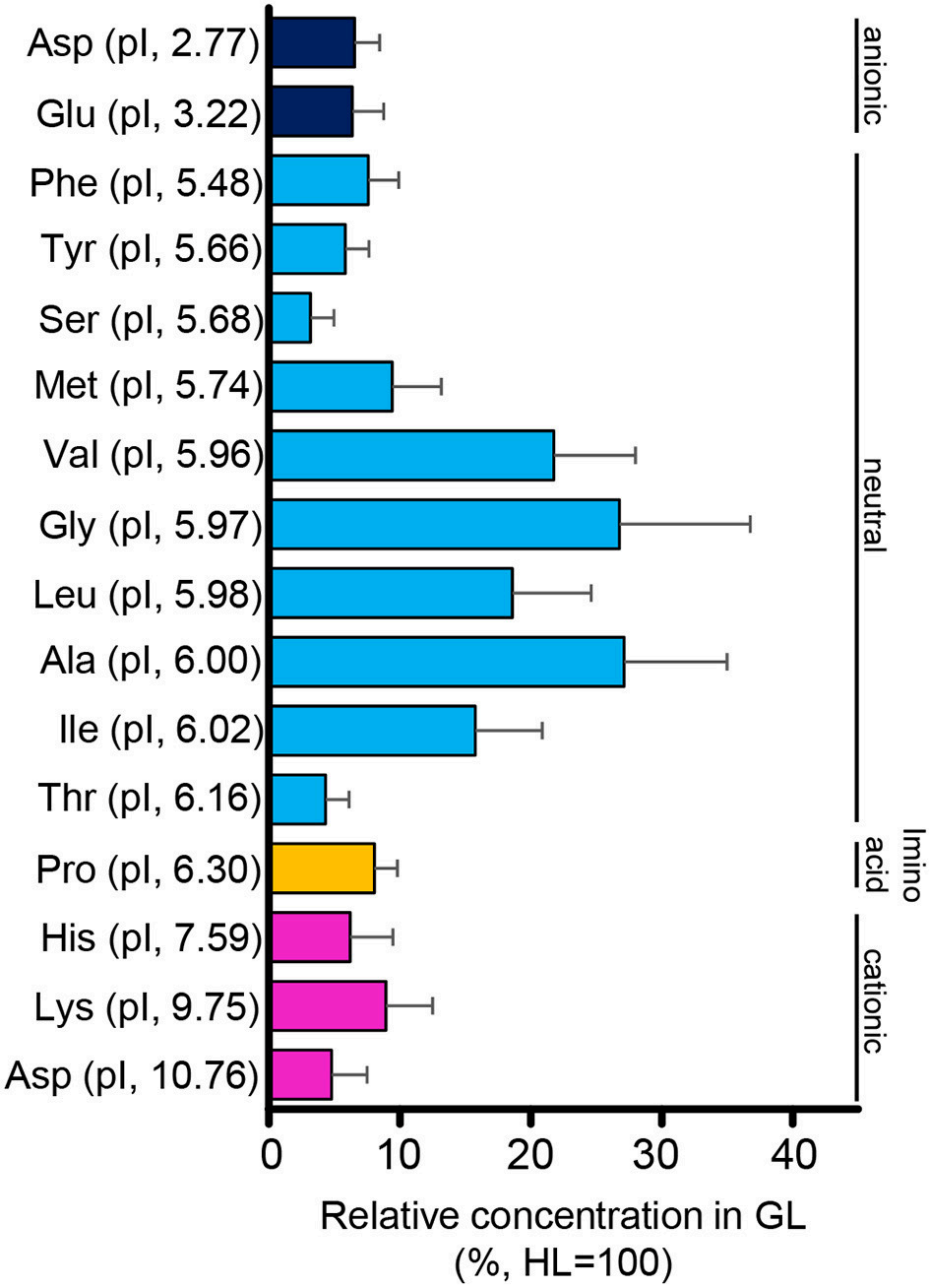
Relative concentrations (%) of amino acids in the intestinal fluid of the GL to that of the HL. Relative concentrations are higher in amino acids with an isoelectric point (pI) close to 6. Error bars indicate the standard error of the mean (SEM).

The protein content in the intestinal fluid was significantly higher in the most proximal part of the intestine (HL) than in the intermediate part (GL), and became lowest in the most distal part (TS) (Figure 4A). Results also showed that the total protein concentration in the solid matter from the chyme did not vary significantly among the HL, GL, and TS (Figure 4B). The results of the free amino acid concentration profile analysis also showed significantly higher concentrations in the HL and significant reductions in the GL and TS (Figure 5). Although considerable amounts of specific neutral amino acids with isoelectric point (pI) around 6 (Val, Gly, Leu, Ala, Ile) still remained in the GL (Figure 6), intestinal absorption of these amino acids were almost completed in the TS (Figures 5K–O).

### Effect of fasting on *slc6a18* and *slc6a19a* expressions

**Figure 7:**
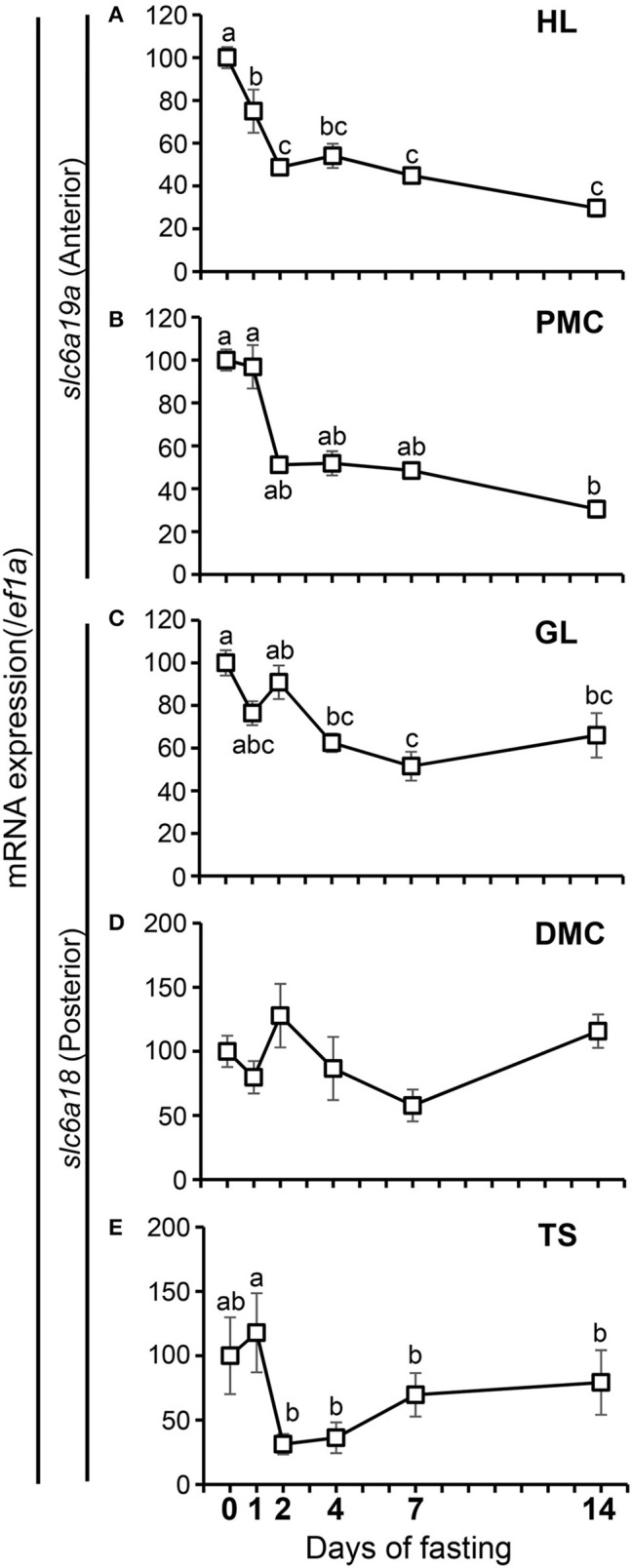
Changes in mRNA expressions of *slc6a19a* in the HL **(A)** and PMC **(B)** and *slc6a18* in the GL **(C)**, DMC **(D)** and TS **(E)** of the intestine in *Mozambique tilapia* following fasting. Expressions were normalized against *ef1a*. Values with different letters are significantly different (*P* < 0.05). Error bars indicate the standard error of the mean (SEM).

The expressions of *slc6a18* and *slc6a19a* in response to short-term and long-term fasting were investigated in the present study (Figure 7). Fish were sampled before the start of fasting (day 0), and during fasting at day 1, day 2, day 4, day 7, and day 14. Results showed that the expression of *slc6a19a* significantly decreased from the initial (day 0) to day 2 of fasting, and then became stable from day 4 to day 14 in the anterior intestine (HL and PMC) (Figures 7A,B). A significant decrease of *slc6a18* expression was observed in the GL (days 4-14) but not in the DMC and TS (Figures 7C–E); however, expression levels of *slc6a18* were lower at day 2 than at day 1 in the TS (Figure 7E).

### Effect of fasting and refeeding on *slc6a18* and *slc6a19a* expressions

**Figure 8:**
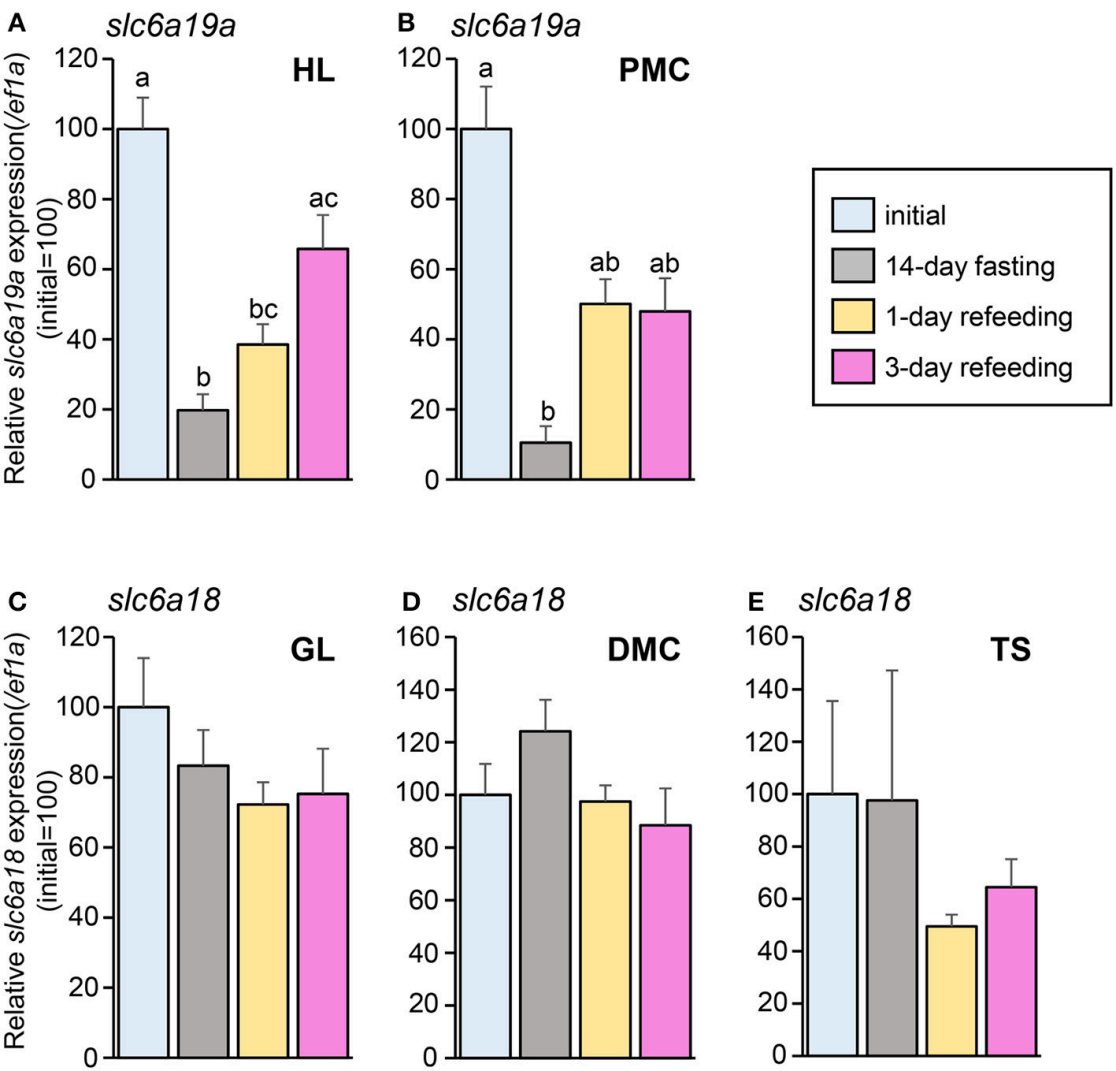
Changes in mRNA expressions of *slc6a19a* in the HL **(A)** and PMC **(B)** and *slc6a18* in GL **(C)**, DMC **(D)**, and TS **(E)** of *Mozambique tilapia* intestine before fasting (initial), at day 14 of fasting, and at day 1 and day 3 of refeeding. Expressions were normalized against *ef1a*. Refeeding stimulates *slc6a19a* expression, while no significant changes are seen in *slc6a18*. Values with different letters are significantly different (*P* < 0.05). Error bars indicate the standard error of the mean (SEM).

In the fasting and refeeding experiment, groups of well-fed fish were fasted for 14 days and then feeding was resumed for 3 days. Fish were sampled before fasting (initial), at the end of 14-day fasting, and at day 1 and day 3 after resumption of feeding. As is the case with the fasting experiment, the expression of *slc6a19a* was decreased in the HL and PMC after 14 days of fasting. Significant increase was observed at day 3 of refeeding in the HL (Figure 8A). In the PMC, the *slc6a19a* expression showed a tendency to be increased at day 1 and day 3 of refeeding, although changes were not statistically significant (Figure 8B). Expression levels of *slc6a18* did not show significant changes after 14-day fasting, and at day 1 and day 3 of refeeding (Figures 8C–E).

## Discussion

### Tissue distribution of *slc6a18* and *slc6a19a*

Slc6a18 and Slc6a19 are the main apical membrane neutral amino acid transporters located at the apical membrane of intestinal and renal epithelial cells in mice (Bröer, 2013; Fairweather et al., 2015). In terrestrial animals, Slc6a18 is specific to the renal epithelial cells, while Slc6a19 is found in both small intestine (duodenum, jejunum, and ileum) and kidney (the proximal tubule and glomerulus) (Bröer et al., 2004; Romeo et al., 2006; Fairweather et al., 2015). Similarly, in the present study, *slc6a19a*, the gene that encode Slc6a19a, was detected in the intestine and kidney of *M. tilapia*, and poorly or not detected in other tissues tested (the brain, gill, heart, liver, esophagus, stomach, rectum, muscle, and skin. Tilapia *slc6a18*, the gene that encodes Slc6a18, was expressed in both intestine and kidney with higher expression in the intestine than in the kidney. This is not the case with the mammalian ortholog, which is expressed only in the kidney. The discrepancy might imply variations in the characteristics of these genes in terrestrial and aquatic animals. Our results might also suggest differences in the mechanisms of amino acid absorption in different species. Moreover, although both *slc6a18* and *slc6a19a* are expressed in the intestine, they showed opposite distribution profiles. The *slc6a18* was specifically expressed in the intermediate (GL) and posterior parts of the intestine (DMC, TS) while *slc6a19a* the expression was restricted to the anterior intestine (HL, PMC). This result shows spatial characteristics of these genes, suggesting possible differences in their absorption function and characteristics.

Whereas, *slc6a19* in aquatic organisms has been reported in previous studies, no information is available on *slc6a18*. Nitzan et al. (2017) reported prominent *slc6a19* expressions in the anterior intestinal sections of *M. tilapia* (*O. mossambicus*) with significant reduction in the posterior intestine. According to the primer sequences, the partial sequence of *slc6a19* analyzed in Nitzan et al. (2017) is most likely to be identical to *slc6a19a* in the present study. In accordance with the estimation, their results resemble the expression pattern of *slc6a19a* in the current experiment. In sea bass, the distribution analysis of *slc6a19* showed high expressions in the intestine and kidney and poor expressions in the stomach, gill, brain, heart, liver, and muscle. *slc6a19* was also found in the pyloric caeca of sea bass (Rimoldi et al., 2015), an organ that is not present in *M. tilapia*. Rimoldi et al. (2015) also showed that *slc6a19* expression is highest in the distal part (segments 8–9) of the intestine, followed by the segments 1–7, and lowest in segment 10. Their findings are different from the results of the present study, where the predominant expression of *slc6a19a* was specifically found in the anterior part of the intestine (HL and PMC). This might be due to the differences in feeding habitats and morphology of the gastrointestinal tract between these two species. Sea bass has a short intestine with no distinct morphological differences among the different regions (Purushothaman et al., 2016). In contrast, tilapia has relatively long intestine, a characteristic of omnivorous/herbivorous fish. Another difference is that *M. tilapia* do not possess the pyloric caeca, an organ involved in digestion and absorption of nutrients in other fishes (Terova et al., 2009). It is highly possible that morphological differences of the gastrointestinal tract could cause variations in the nutrient absorption processes among fish species (Dabrowski et al., 2010). Morphological differences of the digestive tract in different fish species are largely attributed to the diet preferences (i.e., carnivore, herbivore and omnivore) of the organisms (Lassiter and Edwards, 1982; Ferraris and Ahearn, 1984; Buddington et al., 1987).

### Total protein and amino acid concentration profile

The results of the total protein concentration analysis in this experiment showed a decreasing concentration from the HL to GL and TS, which may reflect spatial protein digestion activity in the intestine of tilapia. Protein digestion is likely to occur primarily in the anterior intestine. Similarly, the concentration profiles of free amino acids showed high concentrations in the HL and abruptly reduced concentrations in the GL and TS. The significant decrease of amino acids from the HL to GL and TS suggests that absorption of these amino acids also occurs primarily in the anterior part of the intestine. As to neutral amino acids, the trend coincides with the high expression of *slc6a19a* in the anterior intestine (i.e., HL and PMC). Therefore, like in mammals, *slc6a19a* may play an important role in the absorption of neutral amino acid in the anterior intestine of tilapia. In addition, there remained substantial amounts of amino acids with pI close to 6 in the GL (Val, Gly, Leu, Ala, Ile). It was also observed that both essential (Val, Leu, Ile) and non-essential (Ala, Gly) amino acids remained in the GL. This suggests that the remaining amino acids might not be due to selectivity of absorption between essential and non-essential amino acids. The remaining amino acids in the GL might contribute to transport mechanisms of other amino acids in the intestine. For example, the heterodimeric transporter SLC3A1/SLC7A9 (rBAT/b^0, +^AT), which acts as an absorption mechanism for cationic amino acids, produces efflux of neutral amino acids (Ganapathy et al., 2001; Bröer, 2008b). Moreover, neutral amino acids other than those with pI around 6 were absorbed more efficiently in the anterior intestine (HL and PMC). The affinity of individual amino acids to neutral amino acid transporters may be influenced by the structural and kinetic characteristics of the amino acids and the binding site of the transporters (Preston et al., 1974). In the rabbit ileum, amino acids with unbranched carbon or carbon-sulfide chains showed high affinities to neutral amino acid transporters, whereas affinities were very low in amino acids lacking an alpha amino group, those with alpha-hydrogen substituted by a methyl group, those with tertiary branching in the side chain, and those compounds with either positive or negative charge in the side chain (Preston et al., 1974). In mice, the neutral amino acid transporter Slc6a19 transports amino acids preferentially in the following order: Met, Leu, Ile, Val > Gln, Asn, Phe, Cys, Ala > Ser, Gly, Tyr, Thr, His, Pro > Trp > Lys (Bröer, 2008a). The substrate affinity increases under hyperpolarizing conditions (Bröer, 2008a). These results also imply that absorption of amino acids may be influenced by several factors and may involve more than one mechanism.

In the present study, the absorption of cationic amino acids was almost completed in the anterior intestine, as shown by the very low concentrations in the GL. It has been reported that apical transport of cationic amino acids through heterodimeric antiporter Slc3a1/Slc7a9 (b^0, +^AT/rBAT) partially inhibits the absorption of neutral amino acids. Slc3a1/Slc7a9 is the major cationic transporter in the intestinal epithelial cells and absorbs cationic amino acid from the lumen to the enterocyte in exchange for neutral amino acids (Palacín et al., 2000; Bröer, 2008b). Cationic amino acids have higher affinity to these transporters than the neutral amino acids (Bröer, 2008b). Nitzan et al. (2017) have shown that *slc3a1 and slc7a9* expressions are higher in the anterior and middle intestines than in the posterior intestine of tilapia. Therefore, exchange of cationic and neutral amino acids by Slc3a1/Slc7a9 in the anterior intestine might explain the substantial amounts of some neutral amino acids remaining in the GL. However, these amino acids were diminished in the TS, indicating that neutral amino acid absorption is still active in the posterior intestine. This suggests importance of Slc6a18 which was highly expressed in the posterior intestine. Thus, Slc6a18 might be required to complete absorption of leftovers and reabsorb the neutral amino acids that the antiporter Slc3a1/Slc7a9 releases in exchange for the uptake of cationic amino acids in the intestine.

### Effect of fasting on *slc6a18* and *slc6a19a* expressions

The intestinal epithelial cells are constantly exposed to changing composition of nutrients in the lumen. The amino acid transporters in the intestinal epithelium may be directly or indirectly affected by this condition and may therefore play important roles in maintaining amino acid balance in the body (Steiner and Gray, 1969; Muñíz et al., 1993; Borey et al., 2016). Ferraris and Diamond (1989) reported that luminal nutrients generally act as signals for the transport mechanisms to occur in well-fed animals. In the present study, the effects of nutrient condition on the expressions of neutral amino acid transporters *slc6a18* and *slc6a19a* were examined through fasting. Fasting inhibits normal absorption of required nutrients in the body, thus limiting dietary signals for the transporters. Fasting may activate regulatory mechanisms as a direct response to the diminished nutrient signals, and as indirect responses to factors released by the lack of luminal signals (Ferraris and Carey, 2000). Results of the present study showed that fasting induced downregulation of *slc6a19a* expressions in the HL and PMC. It was observed that drastic decreases of expression levels occurred in the early phase of fasting (until day 2), but no significant changes were observed thereafter. The significant reduction of expression levels at the early phase of fasting may indicate the effect of short-term fasting. It was also observed that while *slc6a19a* expression significantly decreased from day 0 to day 1 in HL, the expression of *slc6a19a* did not change from day 0 to day 1 in PMC. This delay might be related to the difference in the nutrient concentration in the lumen of HL and PMC. Nutrient concentration in the lumen depends on the supply from the stomach. At 1 day after feeding, the stomach becomes empty, as well as the HL which is next to stomach, while remaining undigested food can still be found in PMC; therefore, nutrient concentration in HL was already low while it was still high in PMC. This suggests that *slc6a19a* expression directly reflects nutrient concentration in the lumen, and *slc6a19a* can serve as a good indicator of the status of luminal nutrient condition in fish. A less remarkable decrease in *slc6a18* expression levels was also observed in the GL from day 0 to day 4. However, there was no significant change observed in the DMC. In the TS, although a significant decrease was observed from day 1 to day 2, expression levels after fasting were not different the initial level. These results suggest that the expression of *slc6a18* is more stable under fasting condition than *slc6a19a*. Even in the fasting phase, digestive enzymes and some other proteins are secreted into the lumen at a basal level, and turnover of intestinal epithelia is most likely to occur to maintain the function of the intestine during fasting. Therefore, substantial nutrients derived from the body might be present in the intestinal lumen, and Slc6a18 might contribute to absorb and recycle neutral amino acids in these nutrients. The difference in the response of *slc6a18* and *slc6a19a* to fasting may suggest that absorption of amino acids involves more than one mechanism, and that these amino acid transporters have different roles in the adaptation of tilapia to nutrient condition.

Although several studies have been reported on the effect of fasting on Slc6a19 expression in other species, to the best of our knowledge, no information is still available for Slc6a18. Nitzan et al. (2017) reported different expressions of Slc6a19 at 6, 24, and 72 h after feeding in the anterior, middle and posterior intestine of *M. tilapia*. Their results showed the highest Slc6a19 expression at 6 h after feeding in the middle intestine. Significant reductions at three time points were observed in the posterior intestine. Based on their results, Nitzan et al. (2017) suggested that the main site of Slc6a19 activity was the anterior and middle intestines. Despite of the same species, their result is slightly different from the result of the current experiment where *slc6a19a* was mainly expressed in the anterior intestine (HL, PMC) but not in the middle and posterior intestines. This might be due to the discrepancy in the division of the intestinal segments. In zebrafish, postprandial analysis showed that *slc6a19* expression decreased from 24 to 384 h after feeding (Tian et al., 2015). Spatial and temporal differences in the expression of neutral amino acid transporter Slc6a19 in response to fasting in different species could be accounted for by the difference in the gastrointestinal morphology, which is influenced by dietary preferences and genetic and phenotypic adaptation to diet (Ferraris and Ahearn, 1984; Buddington et al., 1987). The response of *slc6a19a* to chronic fasting is similar to that of the oligopeptide transporter gene *slc15a1* to a long period of fasting. Orozco et al. (2017) reported that *slc15a1a* expression in the anterior intestine of tilapia was decreased at day 4 of fasting and became stable until day 14. In zebrafish, *slc15a1a* also decreased at day 1 and became stable until the day 5 of fasting (Koven and Schulte, 2012). However, *slc15a1a* showed a different expression pattern in the early phase of fasting. Whereas, *slcl6a19a* decreased even at the early phase of fasting, *slc15a1a* expression showed transient increase as observed in tilapia (Orozco et al., 2017) killifish *F. heteroclitus* (Bucking and Schulte, 2012), and sea bass (Hakim et al., 2009). The difference in short-term responses to fasting between *slc6a19a* and *slc15a1a*, both of which are important for amino acid absorption in the anterior intestine, suggests that there are at least two distinct systems to control amino acid absorbing function of the anterior intestine in response to fasting. Orozco et al., (2017) suggested that the temporary upregulation of *slc15a1a* expression is not a direct response to the decreasing luminal nutrient signal, but a primary response to the nutritional status in the body, and not in the lumen, at short-term fasting. This regulatory response of *slc15a1a* might be to maximize absorption in order to accomplish basal nutrient requirement in the body as *slc15a1a* absorbs all types of peptides (cationic, anionic, and neutral). This can also be shown by the lesser downregulation of *slc15a1a* expression in the HL than in the PMC (Orozco et al., 2017). On the other hand, as discussed earlier, *slc6a19a* expression might be directly controlled by the luminal nutrient signals at short-term fasting. At very low amino acid concentration, *slc6a19a* was strongly suppressed both in the HL and PMC, suggesting that Slc6a19a contributes to absorb neutral amino acids available in the intestine, but does not contribute to stimulation of potential nutrient absorption ability to deal with expected feed intake after short-term fasting. These distinct regulatory systems between *slc15a1a* and *slc6a19a* suggest that these nutrient transporters can be specifically manipulated. Although extensive studies are still needed to clarify these observations, the observed difference in regulatory responses of *slc15a1a* and *slc6a19a* might be one of the first steps to a better understanding of these complicated systems.

In terrestrial species, in contrast, the responses of neutral amino acid transporters to fasting and other malnutrition condition showed different patterns. In guinea pigs, starvation stimulates Na^+^-dependent L-alanine transport, as shown by the increased V_max_ of the system (Muñíz et al., 1993). In rats, Na^+^ dependent amino acid transport in the intestine increased during starvation (Newey et al., 1970; Debnam and Thompson, 1984; Waheed and Gupta, 1997). It was also shown, however, that some other Na^+^-dependent amino acid transport systems were not stimulated by starvation (Muñíz et al., 1993). Lis et al. (1972) showed that amino acid uptake increased in the early phase of starvation but decreased after a longer period of fasting. These results suggest that there might be other mechanisms involved in neutral amino acid transport in fasted condition. The increase in neutral amino acid transport in terrestrial species during fasting could be attributed to the increased surface area of the microvilli during fasting, increased fluidity in the brush border membrane of the intestinal epithelium, and hyperpolarization of the brush border membranes during fasting (Newey et al., 1970; Debnam and Thompson, 1984; Muñíz et al., 1993; Waheed and Gupta, 1997; Ferraris and Carey, 2000).

### Effect of fasting and refeeding on *slc6a18* and *slc6a19a* expressions

Despite its ambiguity, the fasting and refeeding method has been increasingly used as a strategy to improve growth and production in aquaculture. On the other hand, its use in the investigations of molecular mechanisms has gained importance as it allows controlled observation of transformations caused by changes in nutritional condition. Fasting and refeeding paradigm has been used to examine effects of nutrient contents on amino acid transporters in fish (Krogdahl and Bakke-McKellep, 2005; Terova et al., 2009; Bucking and Schulte, 2012; Koven and Schulte, 2012; Orozco et al., 2017). In the present study, the downregulation of *slc6a19a* expression caused by fasting was reversed after 3 days of refeeding in HL and PMC. Refeeding supplied dietary nutrients in the lumen, which serve as signals for the transport mechanisms. However, no significant changes were observed in *slc6a18* expression in the GL, DMC and TS during fasting and refeeding. The pattern observed in *slc6a19a* is similar to the response of *slc15a1a* to fasting and refeeding. In *M. tilapia, slc15a1a* was decreased after long-term fasting and increased after refeeding (Orozco et al., 2017). Long-term fasting might induce functional suppression of the digestive organs; inhibited digestion thereby results in delayed stimulation of *slc15a1a* expression after refeeding (Orozco et al., 2017). This explanation might also be applicable to *slc6a19a*. The increased expression of *slc15a1a* at refeeding was lower than the initial level, as in the case of *slc6a19a* in this study. In sea bass, *slc15a1* was decreased during 5 weeks of fasting, and the expression was increased after 4 days of refeeding (Terova et al., 2009). In zebrafish, *slc15a1a* decreased during 5 days of fasting and then increased to a level higher than the initial after refeeding (Koven and Schulte, 2012). Similarly, in killifish, *slc15a1* expression was increased above the pre-fasted level after 1 day and 7 days of refeeding (Bucking and Schulte, 2012). These results show that the transport of amino acids in the intestine may involve more than one pathway and may differ among fish species.

In conclusion, neutral amino acid transporters *slc6a18* and *slc6a19a* in the intestine of tilapia may possess different functional and nutritional characteristics. The *slc6a19a* responds to changes in nutrient condition and showed indications as the major neutral amino acid transporter in the anterior intestine of tilapia. On the other hand, *slc6a18* appeared stable during fasting and refeeding, and might be responsible in the absorption and recycling of amino acids in the posterior intestine. More studies are needed to clarify the amino acid absorption mechanisms and functional characteristics of neutral amino acid transporters *slc6a18* and *slc6a19a*.

## Author contributions

All authors contributed to the design of study. Experiments and analyses were conducted by ZO, SS, and SW. ZO drafted the initial manuscript which was edited by TK and SW.

### Conflict of interest statement

The authors declare that the research was conducted in the absence of any commercial or financial relationships that could be construed as a potential conflict of interest.

## Supplementary material

Figure S1 Expression (copies/μl of template, Mean ± SEM) of the internal standard (*ef1a*) in the tissue distribution analysis.

Figure S2 Expression (copies/μl of template, Mean ± SEM) of the internal standard (*ef1a*) in the HL **(A)**, PMC **(B)**, GL **(C)**, PMC **(D)** and TS **(E)** of the intestine in Mozambique tilapia during the fasting experiment.

Figure S3 Expression (copies/μl of template, Mean ± SEM) of the internal standard (*ef1a*) in the HL **(A)**, PMC **(B)**, GL **(C)**, PMC **(D)** and TS **(E)** of the intestine in Mozambique tilapia during the fasting and refeeding experiment.
