# Supplementary figures and images for: Spatial mRNA Expression and Response to Fasting and Refeeding of Neutral Amino Acid Transporters slc6a18 and slc6a19a in the Intestinal Epithelium of Mozambique tilapia
Source: Front Physiol. 2018 Mar 13;9:212. doi: 10.3389/fphys.2018.00212 (PMC5859172; doi:10.3389/fphys.2018.00212)

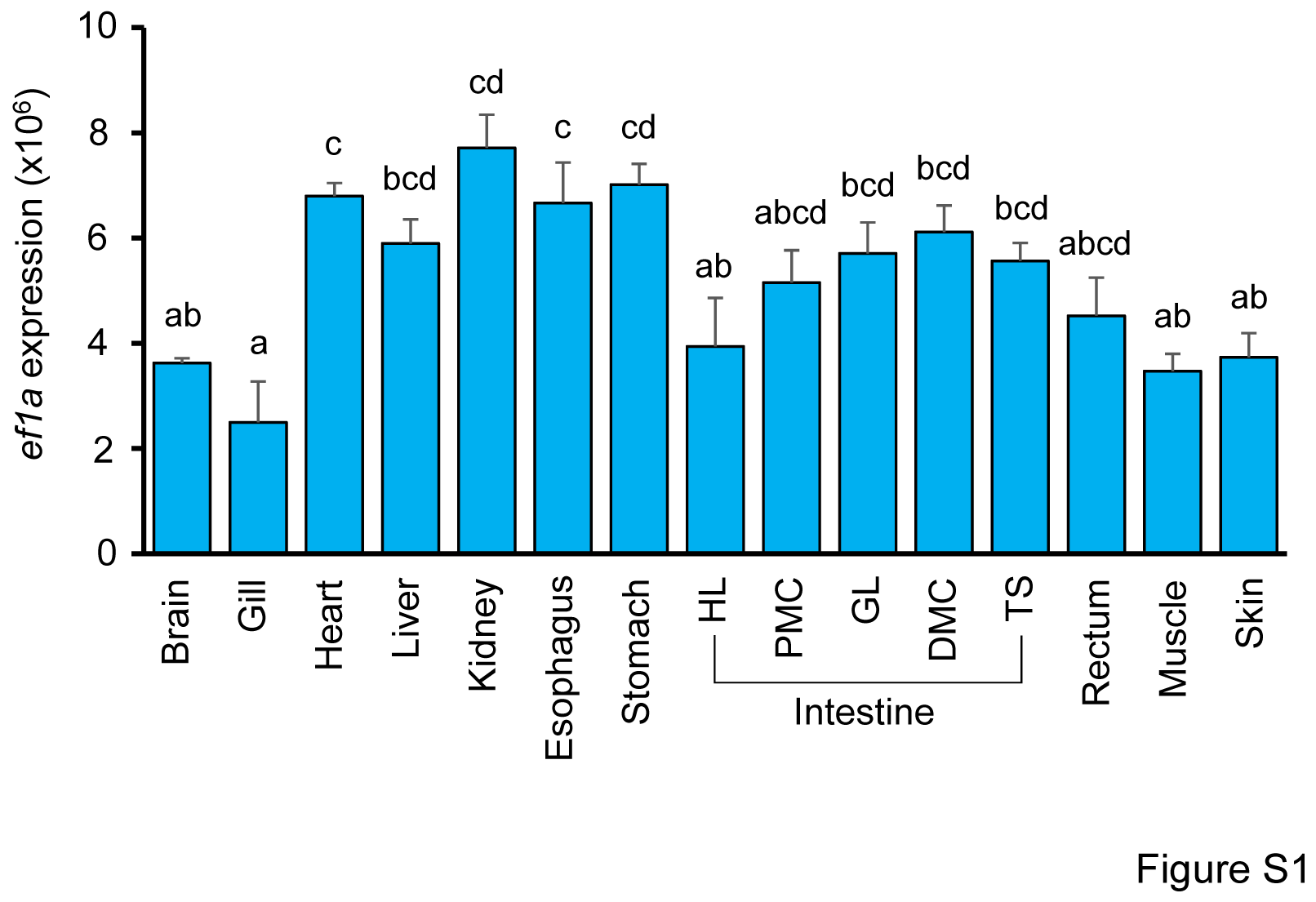

Supplement: Figure S1 — Expression (copies/μl of template, Mean ± SEM) of the internal standard (ef1a) in the tissue distribution analysis. [file Image1.tif]

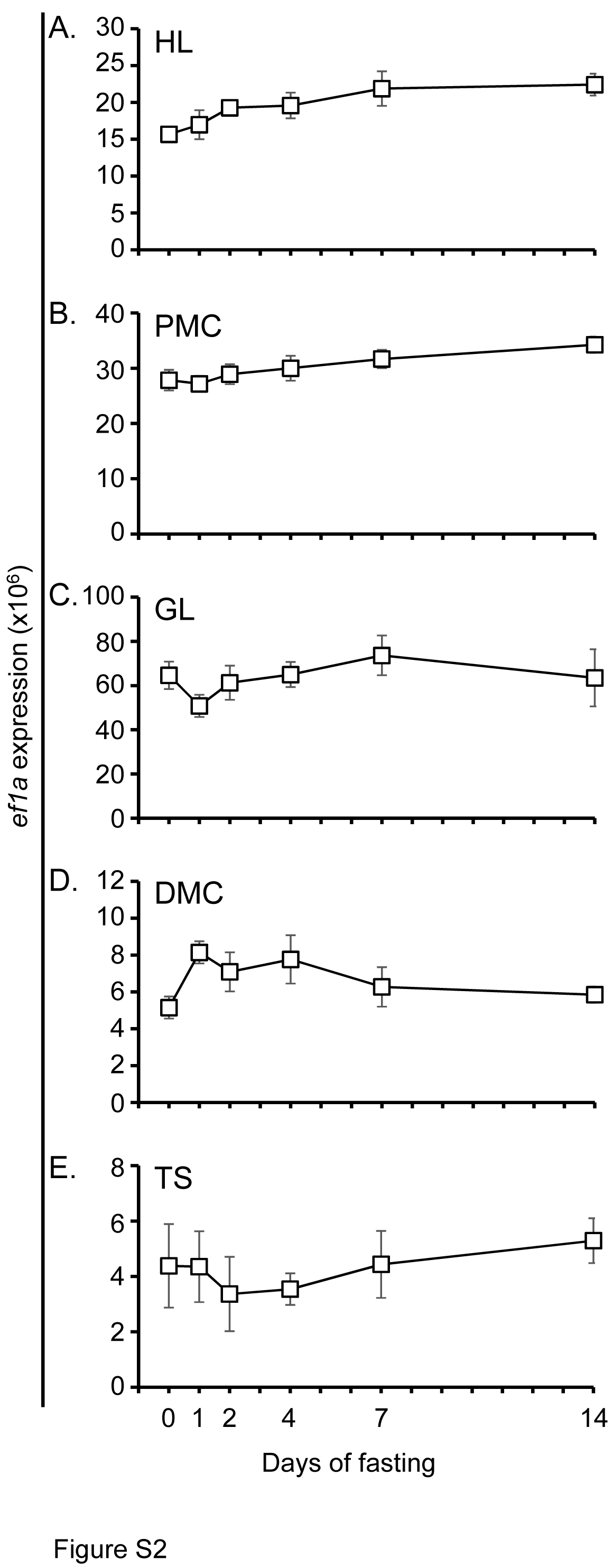

Supplement: Figure S2 — Expression (copies/μl of template, Mean ± SEM) of the internal standard (ef1a) in the HL (A), PMC (B), GL (C), PMC (D) and TS (E) of the intestine in Mozambique tilapia during the fasting experiment. [file Image2.tif]

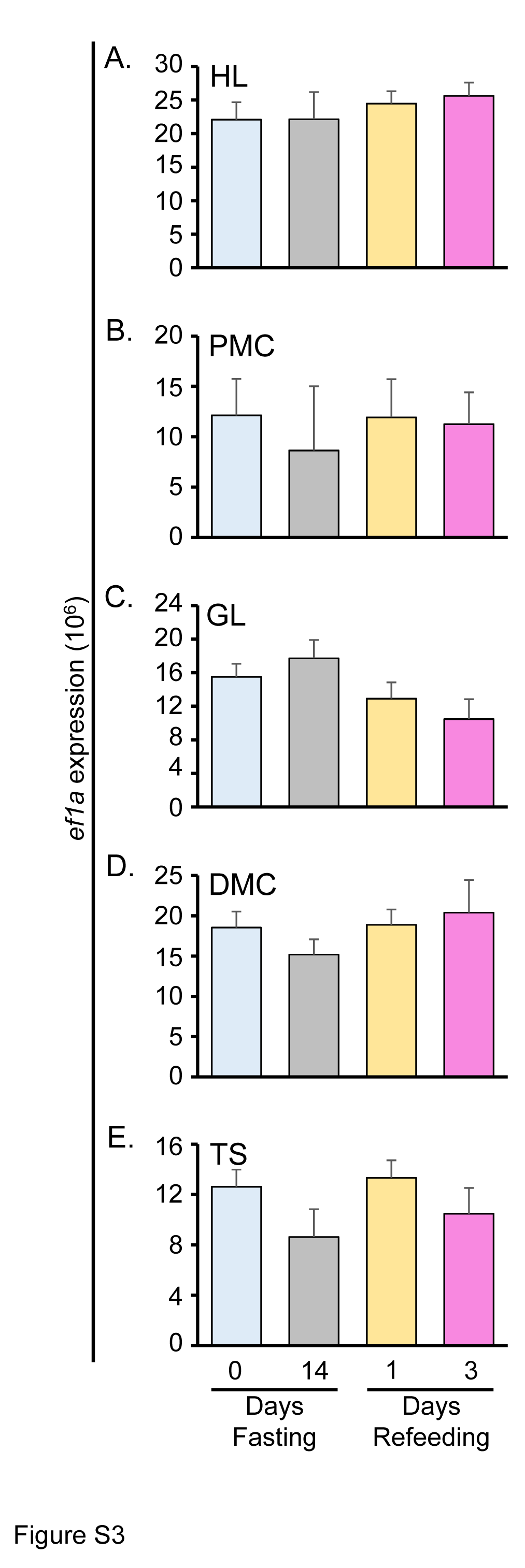

Supplement: Figure S3 — Expression (copies/μl of template, Mean ± SEM) of the internal standard (ef1a) in the HL (A), PMC (B), GL (C), PMC (D) and TS (E) of the intestine in Mozambique tilapia during the fasting and refeeding experiment. [file Image3.tif]
